# RESEARCH NOTE: Exploring the Competence of Various Poultry Species for Cache Valley virus Infection

**DOI:** 10.1016/j.psj.2025.105379

**Published:** 2025-05-30

**Authors:** Krisangel López, John A. Muller, Manette Tanelus, Dawn I. Auguste, William B. Stone, Sally L. Paulson, Amy Rizzo, Chad E. Mire, Albert J. Auguste

**Affiliations:** a Department of Entomology, Fralin Life Science Institute, Virginia Polytechnic Institute and State University, Blacksburg, VA, 24061; b Animal Resources and Care Division, Office of Research, Virginia Polytechnic Institute and State University, Blacksburg, VA, 24061; c United States Department of Agriculture, Agricultural Research Services, National Bio and Agro-defense Facility, Foreign Arthropod-Borne Animal Diseases Research Unit, Manhattan, Kansas, 66502; d Center for Emerging, Zoonotic, and Arthropod-borne Pathogens, Virginia Polytechnic Institute and State University, Blacksburg, VA, 24061

**Keywords:** Cache Valley Virus, Bunyaviruses, Growth Curves, Avian Animal Models

## Abstract

Cache Valley virus (CVV) belongs to the genus *Orthobunyavirus*, and is known to cause severe disease in ruminants, including spontaneous abortions and congenital defects. Previous evidence suggests there is the potential of CVV to infect poultry species due to its wide geographic range, reports of seropositivity in birds for Cholul or Maguari virus (closely related viruses), and isolations of CVV from highly ornithophilic mosquito vectors. To determine CVV’s potential as a disease-causing agent in poultry species, we used two strains from the two recognized genetic lineages of CVV for both our in-vivo and in-vitro studies. We assessed CVV’s growth kinetics in three avian cells lines, including domestic chicken (*Gallus gallus*; DF-1), Japanese quail (*Coturnix coturnix japonica:* QNR/K2*),* and Pekin Duck cells (*Anas platyrhynchos domesticus:* PDE). For the in-vivo studies, we challenged three-day old SPF-chickens (*Gallus gallus*), three-day old ducklings (*Anas platyrhynchos domesticus*), and 14-day old quail (*Coturnix coturnix)* with both CVV strains*.* We found that CVV grew rapidly and to high titers in all three avian cell lines yet failed to induce a symptomatic infection during in-vivo studies. Our data suggests that domestic poultry species are likely not significant contributors to the maintenance of CVV. However, further studies using passerines and mosquito transmission experiments are necessary to determine if CVV has the potential to impact avian species.

## Introduction

Cache Valley virus (CVV) belongs to the genus *Orthobunyavirus* in the family *Peribunyaviridae*. Initially discovered in Cache Valley, Utah, in 1956 (Holden & Hess, 1959), CVV is known to cause severe disease in ruminants, including spontaneous abortions and congenital defects (i.e., arthrogryposis) (Hughes et al., 2023; Waddell et al., 2019). Although rare, CVV has been shown to also cause severe disease in humans, including encephalitis, multiorgan failure, macrocephaly in infants, and death (Hughes et al., 2023). CVV is widespread across North America and multiple outbreaks have been reported across the United States, Canada, and Mexico (Hughes et al., 2023). CVV is also unique in that it has been detected in over 44 mosquito species, and the virus has been isolated from several ornithophilic mosquitoes including *Culex tarsalis, Culex pipiens, Culex quinquefasciatus,* and *Culex restuans* (Waddell et al., 2019).

CVV has been serologically detected or isolated from a wide variety of wildlife and livestock (reviewed in Waddell et al., 2019). Although the primary reservoir host for CVV remains unknown, white-tailed deer *(Odocoileus virginianus*) are suspected as the primary sylvatic host (Hughes et al., 2023). Holden and Hess (1959) challenged 0.5 day-old domestic chicks (*Gallus gallus*) with CVV which resulted in no observed viremia or neutralizing antibodies, suggesting that birds might not be an amplifying host for CVV. However, Belle, Grant, & Griffiths (1966) showed that various species of birds in Jamaica including wild birds and domestic chickens were serologically positive for CVV. Since this study was conducted, the Caribbean and South American strain of CVV has been reclassified as Maguari virus (MAGV) (Hughes et al., 2023). A follow-up survey conducted in the Yucatan peninsula to identify *Orthobunyavirus* presence in poultry and domestic animals (Blitvich et al., 2012), found no antibodies to CVV in either chicken or turkeys. However, they did find antibodies for Cholul virus (CHLV) (a natural recombinant of CVV and Potosi virus) in turkeys (Blitvich et al., 2012). This again prompts the question of the potential agricultural impact that CVV may have in North American poultry, as no true surveys or experiments have been conducted in poultry since the reclassification of MAGV and CHLV.

Altogether, given the seropositivity in birds for MAGV and CHLV which are viruses within the same Bunyamwera serogroup, the isolation of CVV from highly ornithophilic mosquito vectors, the strong association CVV has with livestock, we sought to experimentally assess CVV infection in agriculturally important poultry species by conducting both *in vitro* and *in vivo* studies to evaluate viral growth in avian cell cultures and the progression of disease in various poultry species.

## Materials and Methods

### Viruses and Cell Culture

Strains from two genetic lineages of CVV were used in the study: CVV W08491 from Lineage 1 (provided by Dr. Philip Armstrong at the Connecticut Agricultural Experiment Station) and CVV 4B from Lineage 2). Strains were selected based on their genetic diversity, passage history, and enzootic sources. CVV W08491 was isolated from a pool of *Culex tarsalis* (an ornithophilic species) mosquitoes from North Dakota in 2005 and propagated to a viral titer of 1.09 × 10^7^ plaque forming units (PFU)/mL in Vero-76 cells (African Green Monkey Kidney clone) (ATCC; Manassas, VA, USA). CVV 4B was isolated from an *Aedes japonicus* mosquito pool from Blacksburg, Virginia in 2015, and grown to a viral titer of 7.7 × 10^7^ PFU/mL in Vero-76 cells. The cells were grown to 80% confluent monolayers and an aliquot of each virus was added and observed for cytopathic effect (CPE). Upon observation of 50% CPE, the virus was harvested, typically about 2 days post-infection (DPI). The supernatant was collected and clarified by centrifugation for 10 minutes at 3345 × g at a temperature of 4°C, and then stored at -80°C. Viral titers were estimated by plaque assays on Vero-76 cells as previously described (López et al., 2021).

### CVV growth curves in chicken, duck, and quail cell culture

**Fig. 1:**
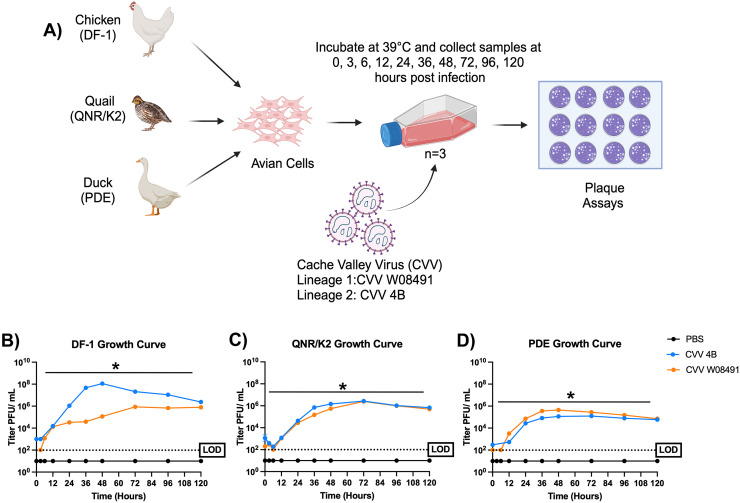
**Cache Valley Virus (CVV) replicates efficiently in avian cell culture systems.** (A) Schematic of the study design used to assess CVV replication in avian cell lines. The three cell lines used are depicted by the corresponding species they were harvested from, and the type of cell used. Flasks were infected with either CVV W08491 (Lineage 1; in orange) or CVV 4B (Lineage 2; in blue), or Negative Control: PBS (Phosphate Buffered Saline; in black) and incubated for 120 hours at 39°C. Samples were run in triplicate and quantified using plaque assay on Vero-76 cells. Viral growth kinetics are shown in (B) DF-1 (*Gallus gallus*), (C) QNR/K2 (*Coturnix coturnix japonica*), and (D) PDE (*Anas platyrhynchos domesticus)* cells. Each data point represents the mean values, and the error bars represent the standard error of the mean. Limit of detection (LOD) is depicted by the dotted line. Statistical significance among the groups was analyzed using repeated measures two-way ANOVA (B-D). Statistically significant values are denoted by *(*p<*0.05). (A) Figure schematics were created using biorender.com.

We used three commercially available avian cells lines; domestic chicken (*Gallus gallus*; DF-1), Japanese quail (*Coturnix coturnix japonica:* QNR/K2*),* and Pekin Duck cells (*Anas platyrhynchos domesticus:* PDE) obtained from Dr. Nisha Duggal at Virginia Tech. The cell lines were grown in T-25 flasks in triplicate to ∼80% confluency and kept at 39°C. Cells were infected with a multiplicity of infection (MOI) of 0.1 PFU/cell. A phosphate buffered saline (PBS) wash (X3) was conducted prior to the initial time point collection to remove any residual virus and supernatant samples were collected for each cell line at 0-, 3-, 6-, 12-, 24-, 36-, 48-, 72-, 96-, and 120-hours post-infection (HPI). All samples were run in triplicate and virus titers quantified using plaque assays as shown in Fig. 1A.

### CVV pathogenesis in chickens, ducks, and quail

**Fig. 2:**
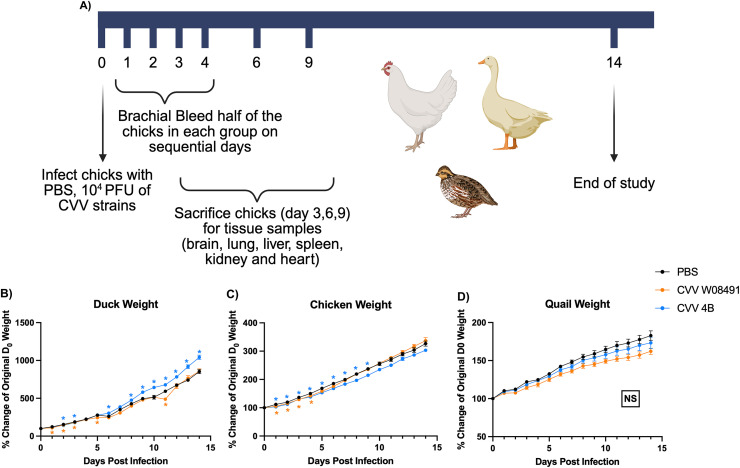
**Cache Valley Virus (CVV) infection does not induce weight loss or mortality in poultry.** (A) Schematic of the *in vivo* study procedure. Three-day old chickens (CVV 4B, CVV W08491, PBS; n=18), two-week old quail (CVV 4B n=11, CVVW08491 n=11, PBS=9), and 3-day old ducks (CVV 4B n=18, CVVW08491 n=17, PBS=16) were subcutaneously inoculated with 10^4^ plaque forming units (PFU) of either CVV W08491 (Lineage 1; in orange), CVV 4B (Lineage 2; in blue), or Negative Control: PBS (Phosphate Buffered Saline; in black), and monitored for 14 days post infection for changes in weight (B-D). Each data point represents the mean values, and the error bars represent the standard error of the mean. Statistical significance among the groups was analyzed using repeated measures two-way ANOVA (B-D). Statistically significant values are denoted by *(*p<*0.05) and color coded to match the experimental infection group. NS denotes non significant values. (A) Figure schematics were created using biorender.com.

Three-day old SPF-chickens (*Gallus gallus*) (n=18) purchased from Charles River laboratories (Wilmington, MA, USA) were subcutaneously (s.c.) inoculated with 10^4^ PFU of either CVV strain (i.e., 4B [n=18] and W08491 [n=18]) or PBS (n=18). Three-day old ducklings (*Anas platyrhynchos domesticus*) purchased from Murray McMurray Hatchery (Webster City, IA) were also s.c. inoculated with 10^4^ PFU of either CVV strain (i.e., 4B [n=18] and W08491 [n=18]) or PBS (n=16). Quail eggs were purchased from AJ Farms LLC (Strasburg, VA, USA). Eggs were incubated for 18 days based on manufacturer recommendations and monitored for hatching. Upon hatch, quail were transferred to isolators and at two weeks old were s.c. inoculated with 10^4^ PFU of either CVV W08491 (n=11), CVV 4B (n=11), or PBS (n=9). All infected birds were monitored daily for symptoms of disease for 14 days, brachially bled on days 1–4, and a subset (n=3) were sacrificed 3, 6, and 9 DPI for tissue collection as described in Fig. 2A. Tissues were stored in culture media (i.e., DMEM containing 2% FBS, 100 units of Penicillin, and 0.1 mg Streptomycin) for virus quantification by plaque assay. Sera collected at the study’s termination day (i.e., 14 DPI) were used for 50% plaque reduction neutralization tests (PRNT_50_) to measure neutralizing antibody responses against respective virus strains (4B and W08491). For the PRNT_50_ assay, sera samples were heat inactivated for one hour at 56°C to inactivate proteins that could interact with the identification of the antibodies of interest. Samples were then diluted using 2% DMEM (components described above) and two-fold serially diluted to generate sera dilutions ranging from 1:20 to 1:640.

A virus only control plate consisting of 100 PFU of virus stock was diluted with equal parts virus stock and 2% DMEM (components described above) and used for each batch of PRNT_50_ assays. Virus-serum mixtures were incubated for one hour at 37°C before being plated in Vero-76 cells. Once plated, a second one-hour incubation followed at 37°C, before a 0.4% agarose overlay (components described above) was added. Plates were fixed 2 DPI using 10% formaldehyde and visualized using 0.2% crystal violet.

### Statistical Analysis

The data is depicted as mean ± Standard Error of the Mean (SEM). To determine differences between groups, samples were analyzed using Logrank Mantel-Cox tests to assess significance among survival (data not shown), and two-way ANOVA with repeated measures to assess significance among growth curve and weight change data. Statistical significance is depicted as a p-value of <0.05. All statistical tests were performed using GraphPad Prism Version 10.2.3

### Ethics Statement

This study was performed under IACUC Protocol 20-071, which was approved by Virginia Tech’s IACUC on 4/9/2020.

## Results and Discussion

In this study, we report the first comprehensive laboratory studies of CVV infection in poultry since the initial study conducted in chickens (Holden & Hess, 1959). In addition to working with chickens, we also explored the potential effects of CVV in two additional poultry species, including domestic ducks and Japanese quail. CVV grew rapidly and to high titers in these avian cell lines yet failed to induce a symptomatic infection in any of the poultry species used here. This data suggests that there is a component of the avian immune system that makes these poultry species resistant to CVV induced disease. A previous study demonstrated that mice lacking the alpha and beta type-1 interferon receptor developed viremia and significant disease when infected with CVV, however, the wildtype/immune-competent mouse model did not (López et al., 2021). This suggests that the avian innate immune response could be playing a significant role in the viruses’ ability to cause infection and disease and could be preventing the virus from spilling over into poultry. Further, studies to assess how the avian immune system might evade CVV infection are warranted to better assess potential transmission and spillover potential.

### CVV grows efficiently in avian cell lines

To determine if CVV can replicate in avian cell cultures, we infected three commercially available avian cell lines (DF-1, QNR/K2, and PDE) with two contemporary strains of CVV (4B and W08491) representing both genetic lineages (Fig. 1A). Our data shows that, regardless of the viral strain used, CVV grows efficiently in all three cell lines. A significant increase in viral titer is observed in all three cell lines by 12 HPI (Fig. 1B-D). In DF-1 cells, CVV replicates exponentially until 48 HPI with a peak viral titer of 8.11 log_10_ (± 0.05 SEM) for CVV 4B and 5.06 log_10_ (± 0.01 SEM) PFU/mL for CVV W08491. CVV plateaus and maintains a titer of 6.38 log_10_ (± 0.1 SEM) for 4B and 5.9 log_10_ (± 0.03 SEM) for CVV W08491 at 120 HPI (Fig. 1B). QNR/K2 shows peak viral growth at 72 HPI, with a viral titer of around 6.43 log_10_ (±0.13 SEM) for CVV 4B, and 6.4 log_10_ (±0.04 SEM) for CVV W08491, which remains consistent until the end of the study (Fig. 1C). In PDE cells, CVV achieved peak titers earlier than the other two cell lines by 36 HPI, however, the overall titers are lower at 4.91 log_10_ (± 0.03 SEM) for CVV 4B and 5.55 log_10_ (±0.02 SEM) for CVV W08491 which remains consistent until the end of the study (Fig. 1D). Although all of the cell lines showed small differences in growth, DF-1 was the only cell type that shows significant growth differences between viral strains, with CVV 4B (Lineage 2) having a four-log difference when compared to the ancestral lineage 1, CVV W08491 (Fig. 1B). This discrepancy could be explained by the immunological differences and cell types within these cell lines (DF-1 and PDE are fibroblasts and QNR/K2 are nueroretina). Previous studies have presented significantly different responses to viral infections even between poultry species within the same taxonomic order (e.g., Pekin duck [*Anas platyrhynchos*] and Muskovy ducks [*Cairina moschata*], Schultz & Magor, 2022). Further studies into the immunological differences and cell types between these cell lines are warranted to explore the differences in viral replication patterns observed in this study.

### CVV does not cause morbidity or mortality in poultry species

To explore the pathogenesis of CVV in poultry species and potential differences in strain-specific pathogenesis, 3-day old chickens, 3-day old ducks, and two-week old quail were subcutaneously inoculated with either CVV W08491 or 4B, or PBS diluent (Fig. 2A). Birds were monitored for 14 DPI and overall, no significant weight loss, signs of disease, (Fig. 2B-2D) or mortality (data not shown) was observed when compared to healthy controls. Differences observed in the weights across the different cohorts are primarily due to differences in sex ratios as we were unable to sex birds prior to the start of the study to ensure all of the groups had an equal number of both males and females. However, birds were sexed at euthanasia. We noted that the CVV 4B infection group in the ducks was disproportionally male causing the 4B group to have a marginally higher growth rate compared to the W08491 infection group and PBS healthy control group. Similarly, the chickens infected with 4B had a disproportionately larger number of females, presenting a reduced growth rate later in the study compared to the W08491 infection group and PBS healthy control group. Nonetheless, birds did not present weight loss or any signs of disease throughout the study, and remained antibody negative at the study’s conclusion, suggesting the absence of a productive infection. To measure viremia in infected birds, blood was collected 1–4 DPI and virus titers quantified by plaque assay. No viremia was detected among any of the six infected CVV groups (data not shown; limit of detection is 100 PFU). To assess serological status in birds at the study’s end, blood was collected 14 DPI and sera harvested for PRNT_50_ testing. CVV failed to induce any detectable neutralizing antibody response in any of the infected birds (limit of detection being 1:20 PRNT_50_ dilution).

Altogether, our results show that CVV grows well in avian cell culture systems but failed to illicit symptomatic infections or neutralizing antibody responses in any of the poultry species studied. These experimental infection data suggests that CVV may be an unlikely pathogen for poultry species. Future studies are needed and should also include a Caribbean and Central/ South American strain of CVV, such as Maguari virus (Groseth et al., 2017), or Cholul virus which is a recombinant strain of CVV and Potosi virus, that has been previously isolated from turkeys (Blitvich et al., 2012). These studies could help determine if there are virus-specific restrictions preventing CVV from causing a systemic and symptomatic infection in birds. Additionally, a previous study conducted in mice, showed that CVV did not produce an infection in immune competent mice, however, adding mosquito saliva to the inoculum elicited a symptomatic infection (Edwards, Higgs, & Beaty, 1998). Future studies are necessary with mosquito saliva in the inoculum or using mosquitoes for direct transmission may help induce a symptomatic infection in these bird species.

Although CVV did not induce disease or neutralizing antibody responses in the poultry species tested here, there remains the possibility that CVV may be able to replicate and cause disease in non-poultry avian species. Belle et al. (1966) showed that various wild passerine species tested serologically positive for CVV. Experimental infections of various passerine species (such as house sparrows, zebra finches or canaries) are needed to determine if passerines can play a role in the maintenance and dispersal of CVV both locally and potentially internationally during long-distance migration (Belle et al., 1966; Reed, Meece, Henkel, & Shukla, 2003).

In summary, our data suggests it is unlikely that domestic poultry species are significant contributors to the maintenance of CVV within agricultural systems or will suffer significant disease as a result of CVV infection. However, further transmission studies, the use of other closely related viruses (i.e., Cholul or Maguari virus), and exploring competence in passerines are necessary to determine if CVV has the potential to expand into other avian species.

## Disclosures

The authors declare that they have no known financial interests or personal relationships that could have appeared to influence the work reported in this paper.
